# Antibiotic use during pregnancy and childhood overweight: A population-based nationwide cohort study

**DOI:** 10.1038/s41598-019-48065-9

**Published:** 2019-08-08

**Authors:** Tine Jess, Camilla S. Morgen, Maria C. Harpsøe, Thorkild I. A. Sørensen, Teresa A. Ajslev, Julie C. Antvorskov, Kristine H. Allin

**Affiliations:** 1 Center for Clinical Research and Prevention, Bispebjerg and Frederiksberg Hospital, The Capital Region, Copenhagen, Denmark; 2 Department of Epidemiology Research, Statens Serum Institut, Copenhagen, Denmark; 3 National Institute of Public Health, University of Southern Denmark, Copenhagen, Denmark; 4 Department of Public Health, Section of Epidemiology, Faculty of Health and Medical Sciences, University of Copenhagen, Copenhagen, Denmark; 5 Department of Clinical Microbiology, Hvidovre Hospital, Copenhagen, Denmark; 6 Novo Nordisk Foundation Center for Basic Metabolic Research, Faculty of Health and Medical Sciences, University of Copenhagen, Copenhagen, Denmark; 7 The Bartholin Institute, Rigshospitalet, Copenhagen, Denmark

**Keywords:** Obesity, Epidemiology, Paediatric research

## Abstract

Studies in mice suggest that early life represents a critical time window, where antibiotics may exert profound and lasting effects on the gut microbiota and metabolism. We aimed to test the hypothesis that prenatal antibiotic exposure is associated with increased risk of childhood overweight in a population-based cohort study. We linked 43,365 mother-child dyads from a nationwide cohort of pregnant women and their offspring to the Danish National Prescription Registry. Linear and logistic regression models were used to examine associations between prenatal exposure to antibiotics and BMI z-score and overweight (including obesity) at age seven and 11 years. Prenatal antibiotic exposure and childhood overweight were both associated with high pre-pregnancy BMI, maternal diabetes, multi-parity, smoking, low socioeconomic status, high paternal BMI, and short duration of breastfeeding. After adjustment for confounders, no associations were observed between prenatal antibiotic exposure and odds of overweight at age seven and 11 years. Whereas no association was observed between broad-spectrum antibiotics and overweight at age 11 years, exposure to broad-spectrum antibiotics was associated with higher odds of overweight at age seven years with an odds ratio of 1.27 (95% CI, 1.05–1.53) for ampicillin and an odds ratio of 1.56 (95% CI, 1.23–1.97) for amoxicillin. As we did not account for underlying infections, the observed associations with early childhood overweight could be explained by confounding by indication. In conclusion, our population-based study suggests that prenatal exposure to narrow-spectrum antibiotics is not associated with overweight in offspring. Exposure to some broad-spectrum antibiotics may increase the odds of overweight in early childhood, but the association does not persist in later childhood.

## Introduction

Antibiotics are widely used during pregnancy. Among all pregnant women in Denmark during years 2000–2010, approximately one third received antibiotics^1^. Antibiotics represent one of the most important environmental influences on microbial ecology. Specifically, it has been shown that oral antibiotics in pregnancy alter the vaginal microbial ecology^2^. The gut microbiota of children is established during the first few years of life, which represents a critical time window, where the gut microbiota is especially vulnerable to external influences, including antibiotics^3^. As neonates acquire their microbes from their mother during delivery^3^, and since several studies have suggested that the gut microbiota contributes to metabolic diseases^4^, it has been hypothesized that exposure to antibiotics during pregnancy alters the composition of the founding microbiota of the neonate and thereby contributes to development of childhood overweight and obesity^3^.

Whereas several studies have examined the association between antibiotics in infancy and overweight^5^, fewer studies have examined whether *prenatal* exposure is associated with higher odds of childhood overweight, and results are conflicting^6–10^. Three studies reported larger body size among children exposed to antibiotics in utero^6–8^. Two included less than 600 participants^6,8^, and the third study did not control for maternal body mass index (BMI)^7^, which is an important risk factor for childhood overweight^11^. A fourth study evaluated BMI at age three years, and did not find an association between antibiotics and childhood BMI^9^, whereas a fifth study of children born back in 1959–1965 reported no association with obesity at age 4 years, but found that repeated prenatal exposure to antibiotics was associated with childhood obesity at age seven years^10^. Thus, statistically well-powered studies with detailed information on antibiotic use as well as on potential confounders are still warranted to determine the impact of prenatal antibiotic exposure on childhood overweight.

The aim of the present study was to test the hypothesis that prenatal exposure to antibiotics is associated with childhood overweight. To address this hypothesis, we linked 43,365 mother-child dyads from a nationwide study of pregnant women and their offspring to the Danish National Prescription Registry and examined the odds of overweight at age seven and 11 years according to antibiotic exposure in utero, while accounting for a range of potential confounders.

## Materials and Methods

### Study population

The present study is based on the Danish National Birth Cohort, a nationwide ongoing study of pregnant women and their offspring. Between 1996 and 2002, women were invited to the cohort at their first antenatal visit at the general practitioner. The average response rate was 60%. The pregnant women participated in four telephone interviews, scheduled to take place in gestational weeks 12 and 30 as well as six and 18 months post-partum. Follow-up was undertaken at age seven and 11 years. A total of 100,418 pregnancies were enrolled in the cohort^12,13^. For the present study, we included mother-child dyads, where the birth was a singleton term live birth with birth weight >500 g and to ensure independency of the observations, we included only the first-born child in case of siblings (n = 81,229). Of these, 43,365 children had information on BMI at age seven and/or 11 years of age.

### Antibiotic exposure

Information on use of systemic antibiotics – anatomical therapeutic chemical code (ATC) J01 – was obtained from the Danish National Prescription Registry which contains information on all prescription drugs dispensed at Danish pharmacies^14^. Use of antibiotics was quantified as courses with a new course defined as a prescription with a minimum of 14 days after the previous prescription of antibiotics. Repeated use of the same type of antibiotics within one month was, however, considered as one course. First, second, and third trimester was defined as day 0 to 91, day 92 to 189, and day 190 to the date of birth. Type of antibiotic was categorized as ever use of (1) beta-lactam antibacterials, penicillins (J01C), (2) sulfonamide and trimethoprim (J01E), and (3) macrolides, lincosamides and streptogramins (J01F). Moreover, antibiotics were categorized into narrow- and broad-spectrum antibiotics as described in Supplementary Table 1. If mothers received both narrow- and broad-spectrum antibiotics they were categorized as having received broad-spectrum antibiotics. Broad-spectrum antibiotics were further sub-divided into the categories (1) ampicillin (J01CA01, J01CA02, and J01CA06) (2) amoxicillin (J01CA04 and J01CR02), and (3) sulphamethizole (J01EB02). In addition to prenatal exposure, we also retrieved information on antibiotic exposure during the first six months of life, which has previously been shown to associate with childhood overweight^5^.

### Outcome

Outcomes were BMI z-score and overweight at age seven and 11 years. Anthropometrics at age seven years were reported by the mother (67%), school doctor, public health nurse, or general practitioner. A later evaluation showed that slightly fewer children were categorized as overweight based on self-reported BMI compared to BMI measured by school doctors. However, no systematic differences in the size of the disagreements by the child’s size were found^15^. The mother or father reported information on weight and height at the 11-year follow-up. BMI was calculated as body weight divided by the squared height in meters (kg/m^2^). If the measurements of height and weight were performed at different dates, BMI was calculated only if the two dates were separated by <92 days. To address skewness in the BMI distributions, the Lambda-Mu-Sigma (LMS) method was used to transform BMI at age seven and 11 years into internal age- and sex-specific z-scores^16,17^. Overweight (including obesity) was defined according to age- and sex-specific criteria defined by the International Obesity Task Force^18^. Thinness grade 2 was defined according to age- and sex-specific criteria defined by the International Obesity Task Force^19^.

### Covariates

Information on maternal age at conception, pre-pregnancy BMI, diabetes (gestational diabetes or diabetes before pregnancy), gestational weight gain, paternal BMI, smoking during pregnancy, parity, socioeconomic status, and duration of breastfeeding was obtained from the interviews during and after pregnancy^11^. Socioeconomic status was based on the highest level of maternal education and occupation. The measure was based on the current or most recent job within six months, or, if the woman was studying, on the educational level^20^. Information on gestational age, child gender, birth mode, and birth weight was obtained from the Danish Medical Birth Registry^21^.

### Statistical analyses

Chi-square tests and t-tests were used to test for differences in cohort characteristics according to maternal exposure to antibiotics during pregnancy and child overweight at age seven and/or 11 years. We used linear regression models to examine differences in BMI z-scores between exposed and unexposed children and logistic regression models to examine odds ratios (ORs) of overweight. Data were analyzed using unadjusted and multivariable-adjusted models. Multivariable-adjustment included maternal age at birth, maternal pre-pregnancy BMI, maternal diabetes (yes/no), smoking during pregnancy (0, 0–10, >10 cigarettes per day), parity (0, 1+), socio-economic status (low, medium, or high status), gestational age at birth, and child gender. These covariates were included based on their association with the exposure and outcome. We excluded mother-child dyads who did not have information on the above-mentioned covariates (n = 5587) implying that the same number of individuals was included in unadjusted and adjusted analyses. In a sensitivity analysis, we additionally adjusted for gestational weight gain, caesarian section (yes/no), and birth weight (these covariates associated with either antibiotics exposure or childhood BMI), duration of any breastfeeding (0.0–19.9; 20.0–31.9; 32.0–39.9; and 40.0–95.0 weeks; this covariate exerts its effect after the exposure), and paternal BMI (information was available only on a subset of the cohort). To examine whether antibiotic exposure during the first six months of life confounded the results we additionally adjusted for this. To examine effect modification, we stratified the analyses by maternal pre-pregnancy BMI (<25, 25–30, or ≥30 kg/m^2^), caesarian section, birth weight (<2500, 2500–3999, or ≥4000 g), and child gender. These variables were chosen based on previous literature demonstrating effect modification on early-life antibiotics and childhood overweight associations^5,7,22,23^. All statistical analyses were performed using the SAS (SAS Institute) version 9.4.

### Ethical approval

Each pregnant woman gave written informed consent at enrolment, also encompassing the later follow-up surveys. The establishment of the cohort was approved under ref. no (KF) 01-471/94 by the Committee on Biomedical Research Ethics. The Danish Data Protection Agency approved the data collection of the cohort, the 7-year follow-up, and the 11-year follow-up.

## Results

A total of 43,365 mother-child dyads were included in the study: 40,810 children had information on BMI at age seven years and 24,461 had information on BMI at age 11 years (Supplementary Figure 1). Compared to 37,864 mothers who were not included in the study due to missing information on childhood BMI, the women who were included were slightly less likely to receive antibiotics, had slightly lower pre-pregnancy BMI, lower prevalence of smoking, higher socioeconomic status, and longer duration of breastfeeding (Supplementary Table 2). At age seven years, 10.0% of the children were overweight and at age 11 years, 9.0% of the children were overweight. For children with information on overweight at both age seven and 11 years (n = 21,906), 51.8% of the children who were overweight at age seven, were also overweight at age 11 years. Among the women included in the present study, 24.8% received antibiotics during pregnancy (Supplementary Table 3), and among those who received antibiotics, 75.4% received only a single course and 76.1% received penicillins (J01C). Exposure to antibiotics in pregnancy was associated with higher pre-pregnancy BMI, higher prevalence of maternal diabetes, multi-parity, higher prevalence of smoking, lower socioeconomic status, higher paternal BMI, and shorter duration of breastfeeding (Table 1). Overweight at age seven or 11 years showed a similar pattern of association and was additionally associated with male gender, higher birth weight, and caesarian section (Table 2). Supplementary Table 4 shows characteristics of the study population according to number of antibiotic courses.

**Table 1:** Characteristics of the study population according to use of antibiotics during pregnancy.

	n	Antibiotics during pregnancy		*P* value
		No	Yes	
		Mean ± SD or n (%)	Mean ± SD or n (%)	
Maternal age at birth, years	43,365	30.2 ± 4.2	30.1 ± 4.3	0.02
Maternal pre-pregnancy BMI, kg/m^2^	41,057	23.3 ± 3.9	23.8 ± 4.4	<0.001
Maternal diabetes, n (%)	41,871	254 (0.8)	120 (1.2)	0.001
Smoking in pregnancy	41,583			<0.001
Non-smokers		24,332 (77.8)	7513 (73.0)	
1–10 cigarettes per day		5487 (17.5)	2086 (20.3)	
>10 cigarettes per day		1468 (4.7)	697 (6.8)	
Parity ≥ 1, n (%)	41,662	15,292 (48.8)	5686 (55.1)	<0.001
Family education/occupational class, n (%)	39,739			<0.001
Highest level		21,465 (71.7)	6562 (66.9)	
Middle level		7786 (26.0)	2898 (29.6)	
Lowest level		684 (2.3)	344 (3.5)	
Gestational age at birth, days	43,365	281.9 ± 8.8	281.9 ± 9.0	0.71
Child gender, girls, n (%)	43,365	15,983 (49.0)	5229 (48.7)	0.53
Weekly gestational weight gain, kg	34,994	0.38 ± 0.1	0.37 ± 0.1	0.03
Paternal BMI, kg/m^2^	32,999	25.0 ± 3.1	25.2 ± 3.2	<0.001
Birth weight, kg	43,365	3.6 ± 0.5	3.6 ± 0.5	0.27
Cesarean section, n (%)	43,365	4516 (13.9)	1489 (13.9)	0.98
Breastfeeding, n (%)	30,436			<0.001
0.0–19.9 weeks		5665 (24.7)	2326 (31.2)	
20.0–31.9 weeks		4196 (18.3)	1325 (17.8)	
32.0–39.9 weeks		5421 (23.6)	1588 (21.3)	
40.0–95.0 weeks		7702 (33.5)	2213 (29.7)

**Table 2:** Characteristics of the study population according to child overweight at seven or 11 years of age.

	n	Overweight at seven or 11 years of age		*P* value
		No	Yes	
		Mean ± SD or n (%)	Mean ± SD or n (%)	
Maternal age at birth, years	43,365	30.2 ± 4.2	30.1 ± 4.3	0.22
Maternal pre-pregnancy BMI, kg/m^2^	41,057	23.1 ± 3.8	25.6 ± 5.0	<0.001
Maternal diabetes, n (%)	41,871	306 (0.8)	68 (1.3)	<0.001
Smoking in pregnancy	41,583			<0.001
Non-smokers		28,362 (77.7)	3483 (68.4)	
1–10 cigarettes per day		6384 (17.5)	1189 (23.3)	
>10 cigarettes per day		1743 (4.8)	422 (8.3)	
Parity ≥1, n (%)	41,662	18,214 (49.8)	2764 (54.2)	<0.001
Family education/occupational class, n (%)	39,739			<0.001
Highest level		25,086 (71.9)	2941 (60.9)	
Middle level		9000 (25.8)	1684 (34.9)	
Lowest level		823 (2.4)	205 (4.2)	
Gestational age at birth, days	43,365	281.9 ± 8.8	282.1 ± 9.0	0.06
Child gender, girls, n (%)	43,365	18,448 (48.5)	2764 (52.1)	<0.001
Weekly gestational weight gain, kg	34,994	0.38 ± 0.13	0.38 ± 0.16	0.36
Paternal BMI, kg/m^2^	32,999	24.9 ± 3.0	26.5 ± 3.6	<0.001
Birth weight, kg	43,365	3.6 ± 0.5	3.8 ± 0.5	<0.001
Cesarean section, n (%)	43,365	5179 (13.6)	826 (15.6)	<0.001
Breastfeeding, n (%)	30,436			
0.0–19.9 weeks		6760 (25.3)	1231 (32.9)	<0.001
20.0–31.9 weeks		4892 (18.3)	629 (16.8)	
32.0–39.9 weeks		6239 (23.4)	770 (20.6)	
40.0–95.0 weeks		8807 (33.0)	1108 (29.6)	

Overweight (including obesity) was defined according to age- and sex-specific criteria defined by the International Obesity Task Force.

### Antibiotics and childhood overweight

**Figure 1:**
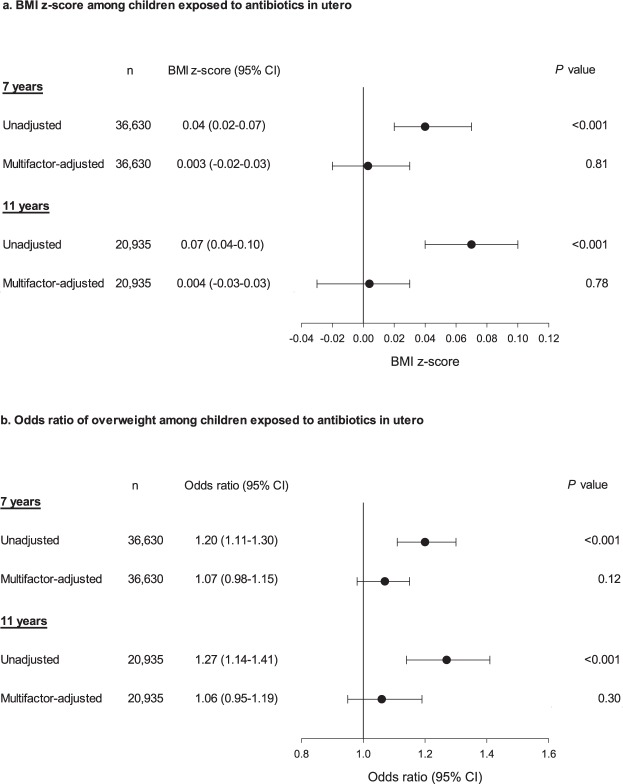
Association between prenatal antibiotics and childhood BMI. (**a**) BMI z-score among children of mothers who received antibiotics vs. children of mothers who did not (**b**) Odds ratio of childhood overweight (including obesity) among children of mothers who received antibiotics vs. children of mothers who did not.

After multivariable-adjustment no association was observed between exposure to antibiotics during pregnancy and BMI z-score at age seven years: 0.003 (95% CI, −0.02–0.03), *P* = 0.81 or 11 years: 0.004 (95% CI, −0.03–0.03), *P* = 0.78 (Fig. 1). When analyzing ORs of overweight, we observed a comparable pattern. Thus, the multivariable-adjusted OR of overweight at age seven years was 1.07 (95% CI, 0.98–1.15), *P* = 0.12 and the OR of overweight at age 11 years was 1.06 (95% CI, 0.95–1.19), *P* = 0.30 (Fig. 1). Additional adjustment for gestational weight gain, breastfeeding, caesarian section, birth weight, and paternal BMI resulted in similar findings (data not shown).

#### Robustness

Of the covariates included in the multivariable model, adjustment for pre-pregnancy BMI attenuated the ORs most (Supplementary Table 5). Exclusion of children with thinness resulted in similar ORs of overweight (data not shown). When analyzing odds of obesity, no association was observed after multivariable-adjustment (Supplementary Table 6). When stratifying on maternal pre-pregnancy BMI, caesarian section, birth weight, and child gender, we observed no association between exposure to antibiotics and overweight at age seven or 11 years in any strata after multivariable-adjustment (*P* for interactions > 0.05) (Table 3).

**Table 3:** Odds of overweight at age seven and 11 years associated with prenatal antibiotics stratified by maternal pre-pregnancy BMI, cesarean section, birth weight, and sex.

	n	Unadjusted		Multivariable-adjusted	
		OR (95% CI)	*P* value	OR (95% CI)	*P* value
Overweight at age seven years					
Maternal pre-pregnancy BMI					
Normal weight (<25 kg/m^2^)	27,238	1.15 (1.04–1.28)	0.007	1.08 (0.97–1.20)	0.17
Overweight (25–30 kg/m^2^)	6844	1.12 (0.96–1.29)	0.14	1.08 (0.93–1.25)	0.32
Obese (≥30 kg/m^2^)	2548	1.11 (0.91–1.35)	0.31	1.03 (0.84–1.26)	0.78
Caesarean section					
No	31,640	1.21 (1.11–1.31)	<0.001	1.08 (0.99–1.17)	0.1
Yes	4990	1.17 (0.96–1.44)	0.12	0.99 (0.80–1.23)	0.95
Birth weight					
<2500 g	335	1.93 (0.87–4.31)	0.11	1.53 (0.63–3.68)	0.35
2500–3999 g	27,423	1.17 (1.06–1.28)	0.002	1.04 (0.94–1.14)	0.47
≥4000 g	8872	1.24 (1.08–1.42)	0.002	1.11 (0.97–1.28)	0.13
Gender					
Girl	17,804	1.15 (1.03–1.28)	0.01	1.02 (0.92–1.14)	0.69
Boy	18,826	1.27 (1.14–1.41)	<0.001	1.12 (0.99–1.25)	0.06
**Overweight at age 11 years**					
Maternal pre-pregnancy BMI					
Normal weight (<25 kg/m^2^)	15,897	1.15 (0.98–1.34)	0.08	1.05 (0.90–1.23)	0.52
Overweight (25–30 kg/m^2^)	3717	1.10 (0.90–1.33)	0.36	1.06 (0.87–1.29)	0.57
Obese (≥30 kg/m^2^)	1321	1.31 (1.01–1.70)	0.04	1.13 (0.86–1.48)	0.38
Cesarean section					
No	18,050	1.26 (1.12–1.41)	<0.001	1.07 (0.94–1.20)	0.31
Yes	2885	1.34 (1.02–1.75)	0.04	1.04 (0.77–1.39)	0.82
Birth weight					
<2500 g	186	0.74 (0.23–2.35)	0.6	0.49 (0.12–1.91)	0.3
2500–3999 g	15,655	1.22 (1.07–1.40)	0.003	1.03 (0.90–1.18)	0.67
≥4000 g	5094	1.36 (1.13–1.63)	0.001	1.15 (0.95–1.40)	0.15
Gender					
Girl	10,472	1.23 (1.05–1.43)	0.009	1.04 (0.89–1.23)	0.6
Boy	10,463	1.31 (1.13–1.51)	<0.001	1.08 (0.92–1.26)	0.35

Overweight (including obesity) was defined according to age- and sex-specific criteria defined by the International Obesity Task Force. Multivariable-adjusted ORs are adjusted for maternal age at birth, maternal pre-pregnancy BMI, maternal diabetes, maternal smoking, parity, socio-economic status, gestational age at birth, and child gender.

### Dose, time, and type of antibiotics and childhood overweight

After multivariable-adjustment, we did not observe any associations between one course, two courses, or more than three courses of antibiotics and overweight at age seven or 11 years (Table 4). When studying timing of exposure, only exposure to antibiotics in the second trimester was associated with overweight at age seven years (OR, 1.12; 95% CI, 1.01–1.25).

**Table 4:** Odds of overweight at age seven and 11 years according to exposure to antibiotics.

	n	Unadjusted		Multivariable-adjusted	
		OR (95% CI)	*P* value	OR (95% CI)	*P* value
**Overweight at age seven years**					
No. of antibiotics courses					
0	27,606	1		1	
1	6821	1.18 (1.08–1.28)	<0.001	1.08 (0.99–1.18)	0.10
2	1615	1.34 (1.14–1.56)	<0.001	1.13 (0.97–1.33)	0.12
≥3	588	1.11 (0.85–1.45)	0.44	0.79 (0.59–1.04)	0.09
Use of antibiotics according to trimester					
No use in pregnancy	27,606	1		1	
First trimester	3295	1.17 (1.04–1.32)	0.008	1.02 (0.90–1.15)	0.76
Second trimester	3943	1.28 (1.16–1.43)	<0.001	1.12 (1.01–1.25)	0.04
Third trimester	3588	1.15 (1.03–1.29)	0.01	0.99 (0.88–1.11)	0.86
Type of antibiotics					
No antibiotics	27,606	1		1	
Penicillins; beta-lactam antibacterials	6903	1.22 (1.12–1.32)	<0.001	1.07 (0.98–1.17)	0.15
Sulfonamides and trimethoprim	1485	1.06 (0.89–1.27)	0.49	0.95 (0.79–1.13)	0.54
Macrolides, lincosamides & streptogramins	865	1.23 (0.99–1.52)	0.06	1.10 (0.88–1.37)	0.41
Narrow- or broad-spectrum antibiotics					
No antibiotics	27,606	1		1	
Narrow-spectrum antibiotics	5957	1.13 (1.03–1.24)	0.009	0.99 (0.90–1.09)	0.89
Broad-spectrum antibiotics	3067	1.34 (1.20–1.51)	<0.001	1.20 (1.07–1.36)	0.002
Type of broad-spectrum antibiotics					
No antibiotics	27,606	1		1	
Sulfamethizole	1480	1.07 (0.90–1.27)	0.46	0.95 (0.79–1.14)	0.57
Ampicillin	1094	1.43 (1.20–1.72)	<0.001	1.27 (1.05–1.53)	0.01
Amoxicillin	574	1.76 (1.40–2.22)	<0.001	1.56 (1.23–1.97)	<0.001
**Overweight at age 11 years**					
No. of antibiotics courses					
0	15,949	1		1	
1	3789	1.20 (1.07–1.36)	0.002	1.05 (0.93–1.19)	0.42
2	891	1.29 (1.04–1.61)	0.02	0.997 (0.79–1.26)	0.98
≥3	306	2.06 (1.51–2.81)	<0.001	1.33 (0.95–1.87)	0.1
Use of antibiotics according to trimester					
No use in pregnancy	15,949	1			
First trimester	1790	1.39 (1.19–1.63)	<0.001	1.16 (0.98–1.37)	0.08
Second trimester	2199	1.31 (1.13–1.52)	<0.001	1.06 (0.91–1.24)	0.46
Third trimester	1953	1.31 (1.12–1.53)	<0.001	1.06 (0.90–1.25)	0.48
Type of antibiotics					
No antibiotics	15,949	1			
Penicillins; beta-lactam antibacterials	3806	1.29 (1.15–1.45)	<0.001	1.06 (0.94–1.20)	0.32
Sulfonamides and trimethoprim	825	1.13 (0.89–1.43)	0.32	0.94 (0.73–1.20)	0.6
Macrolides, lincosamides & streptogramins	471	1.40 (1.05–1.87)	<0.001	1.18 (0.87–1.59)	0.3
Narrow- or broad-spectrum antibiotics					
No antibiotics	15,949	1		1	
Narrow-spectrum antibiotics	3320	1.32 (1.17–1.49)	<0.001	1.11 (0.97–1.26)	0.12
Broad-spectrum antibiotics	1666	1.17 (0.99–1.39)	<0.001	0.96 (0.80–1.16)	0.69
Type of broad-spectrum antibiotics					
No antibiotics	15,949	1		1	
Sulfamethizole	822	1.13 (0.89–1.44)	0.31	0.94 (0.73–1.21)	0.65
Ampicillin	569	1.18 (0.89–1.56)	0.25	0.96 (0.71–1.29)	0.77
Amoxicillin	314	1.18 (0.81–1.72)	0.38	0.90 (0.60–1.34)	0.6

Overweight (including obesity) was defined according to age- and sex-specific criteria defined by the International Obesity Task Force. Multivariable-adjusted ORs are adjusted for maternal age at birth, maternal pre-pregnancy BMI, maternal diabetes, maternal smoking, parity, socio-economic status, gestational age at birth, and child gender.

We did not observe an association between exposure to penicillins, sulphonamides and trimethoprim, macrolides, lincosamides, streptogramins, or narrow-spectrum antibiotics and overweight at age seven or 11 years (Table 4). However, exposure to broad-spectrum antibiotics was associated with higher odds of overweight at age seven years after multivariable adjustment: OR 1.20 (95% CI, 1.07–1.36), *P* = 0.002 (Table 4). The most widely used broad-spectrum antibiotics were sulfamethizole, ampicillin, and amoxicillin. Whereas sulfamethizole was not associated with overweight at age seven years, exposure to ampicillin and amoxicillin were associated with ORs of overweight of 1.27 (95% CI, 1.05–1.53) and 1.56 (95% CI, 1.23–1.97), respectively. Associations between overweight at age seven years and prenatal exposure to broad-spectrum antibiotics, ampicillin, and amoxicillin persisted when adjusting for exposure to antibiotics during the first six months of life (Supplementary Table 7). No association was observed between the broad-spectrum antibiotics sulfamethizole, ampicillin, or amoxicillin and overweight at age 11 years.

#### Robustness

When evaluating BMI z-score instead of OR of overweight, broad-spectrum antibiotics and ampicillin were also associated with higher BMI z-score at age seven years (data not shown).

To examine whether selection bias could explain the lack of an association between broad-spectrum antibiotics and overweight at age 11 years, we excluded mother-child dyads that did not have information on BMI at both age seven and 11 years, and results remained similar (data not shown). Supplementary Table 8 shows characteristics of mother-child dyads with and without information on BMI at age 11 years.

## Discussion

Based on 43,365 mother-child dyads from a nationwide study of pregnant women and their offspring, we found that in general, exposure to antibiotics in utero was not associated with higher odds of overweight at age seven or 11 years after appropriate adjustment for confounders. Exposure to broad-spectrum antibiotics was associated with higher odds of overweight at age seven years, but no association was observed with overweight at age 11 years.

Considering the widespread use of antibiotics in pregnancy^1^, it is crucial to elucidate any harmful effects of prenatal antibiotics on the offspring. As neonates acquire their microbes from their mother during delivery^3,24,25^, prenatal antibiotics may not only disturb the maternal microbiota but also the founding microbiota of the newborn and thus the establishment of a healthy and resilient microbiota. In support of this hypothesis, a previous study demonstrated that intrapartum antibiotics were associated with alterations in the composition and richness of the infant gut microbiota^26^. Several studies have suggested that the gut microbiota may be involved in development of metabolic diseases^27^, and consequently it has been speculated that antibiotics, through their influence on the gut microbiota, may contribute to the development of overweight in humans^4^. Moreover, it has been suggested that early life represents a critical time window during which antibiotics have an especially profound and lasting impact on the gut microbiota and consequently metabolism. Accordingly, a previous study showed that male mice that were exposed to low-dose penicillin had increased fat mass and higher hepatic expression of genes involved in adipogenesis in the liver when penicillin was administered to their mothers shortly before birth and during the weaning period compared to administration post weaning^28,29^. Notably, this study also showed that the growth-promoting phenotype could be transferred to germ-free mice via fecal transplants suggesting that the metabolic effects of antibiotics may be attributable to effects on the gut microbiota^28^. Overall, we did not find an association between exposure to antibiotics in utero and higher odds of overweight. Ampicillin and amoxicillin were however, both associated with higher odds of overweight at age seven years, whereas no association was observed for sulfamethizole; an observation which may be attributed to the different antimicrobial spectrum of these antibiotics.

Our finding of no association between prenatal antibiotics exposure in general and childhood overweight is in contrast to three studies, which reported higher BMI among children exposed to prenatal antibiotics^6–8^. These three studies were however, limited by either sample size^6,8^ or lack of control for maternal BMI^7^. Also, differences in prevalence of overweight and pattern of use of antibiotics may explain the conflicting results. Especially, the study by Cassidy-Bushrow *et al*. is not directly comparable to our study as overweight/obesity was evaluated at age two years; as the prevalence of overweight/obesity was much higher, and as two times as many women were treated with antibiotics in that study^6^. On the other hand, our results are in accordance with a previous study evaluating BMI at age three years^9^, which also found no association between prenatal exposure to antibiotics and childhood overweight. In contrast to our findings of no dose-response relationship between number of antibiotic courses and childhood overweight, a study of children born from 1959–1965 reported an association between repeated prenatal exposure to antibiotics and childhood obesity at age seven years^10^. Notably, however, the use of broad-spectrum antibiotics in this cohort was twice as high as in the present cohort^10^.

Compared to the general Danish population, children in the present cohort had a lower prevalence of overweight. Data from the Danish National Survey of Diet and Physical Activity (DANSDA) in 2000–2002, 2003–2004, and 2005–2008 shows that the prevalence estimate of overweight (including obesity) in 4–14-year-old boys was 13% in the 2000–2002 examination and 22% in the 2005–2008 examination^30^. For girls, the corresponding estimates were 18% and 16%. This indicates that the present study, as most observational studies of general populations, represents a slightly healthier population than the general population, and accordingly, the results may not be directly transferable to populations with higher proportions of overweight and obesity.

In our study, exposure to prenatal antibiotics was associated with a range of covariates that also associated with higher odds of childhood overweight. Importantly, we observed an association between prenatal antibiotics and childhood overweight in un-adjusted analyses, but after multivariable-adjustment only exposure to broad-spectrum antibiotics was associated with higher odds of childhood overweight at age seven years. It is unclear why we observed an association between prenatal use of broad-spectrum antibiotics and overweight at age seven but not at age 11 years. Firstly, as mothers of children who had information on BMI at age 11 years were generally healthier than mothers of children who did not have this information, selection bias could influence our results; however, results remained similar when analysis was restricted to children who participated in both examinations. Secondly, the effect observed at age seven years may attenuate over time and thus no longer be present at age 11 years, and thirdly, the association observed at age seven years may simply be a chance finding.

Major strengths of the present study include our thorough adjustment for potential confounders, the appreciable sample size, and the long-term follow-up. Potential limitations must also be acknowledged. First, the most important confounding in any pharmaco-epidemiological study is confounding by indication, in this case, confounding due to the underlying infections. Indeed, it has been suggested that associations between exposure to antibiotics in childhood and childhood overweight actually reflect a relationship between infections (treated with antibiotics) and weight gain^31^. However, we could not retrieve information on indications for antibiotic prescriptions precluding assessment of the role of infections. Controlling for confounding by indication usually further attenuates any observed associations with medications under study. Thus, had we been able to account for underlying infections, some of the associations with antibiotics use observed would likely have been further attenuated. Second, although we retrieved information about timing, dosing, and type of antibiotics prescribed outside hospitals, we did not have information about antibiotics given during hospitalizations. However, as we did observe an association between prenatal antibiotics and childhood overweight before taking confounders into account, it seems unlikely that missing information about in-hospital antibiotics should account for our findings of no association after multivariable-adjustment. Third, childhood weight and height were self-reported, which may influence the precision of our estimates. Lastly, our study results are based on a slightly healthier segment of the study population who also had a slightly lower exposure to antibiotics than those not included. Further, the regression analyses were based on those who had complete information on all potential confounders. Although we cannot exclude selection bias, we assume that the lack of information on single confounders occurs at random. Nevertheless, our results may not pertain to populations that are unhealthier than the current study population.

## Conclusion

Our nationwide study of more than 40,000 mother-child pairs followed prospectively suggests that prenatal exposure to narrow-spectrum antibiotics is not associated with overweight in offspring. Exposure to some broad-spectrum antibiotics may be associated with higher odds of overweight in early childhood, but not in later childhood. As we did not account for underlying infections, the observed association with early childhood overweight could be explained by confounding by indication. Generally, our results provide reassuring information for clinicians treating pregnant women.

## Supplementary information

Supplementary Material

## Data Availability

The present study is based on data from the Danish National Birth Cohort and the Danish National Prescription Registry. Guidelines to application for access to data from the Danish National Birth Cohort can be found here: https://www.dnbc.dk/access-to-dnbc-data. The Danish National Prescription Registry can be accessed through application to and approval from the Danish Data Protection Agency and the Danish Health Data Authority (https://sundhedsdatastyrelsen.dk/da/forskerservice/ansog-om-data) where the purpose and the feasibility of the intended analysis should be accounted for.

## References

[1] Broe A, Pottegard A, Lamont RF, Jorgensen JS, Damkier P (2014). Increasing use of antibiotics in pregnancy during the period 2000-2010: prevalence, timing, category, and demographics. BJOG.

[2] Stokholm J (2014). Antibiotic use during pregnancy alters the commensal vaginal microbiota. Clin Microbiol Infect.

[3] Cox LM, Blaser MJ (2015). Antibiotics in early life and obesity. Nat Rev Endocrinol.

[4] Mikkelsen KH, Allin KH, Knop FK (2016). Effect of antibiotics on gut microbiota, glucose metabolism and body weight regulation: a review of the literature. Diabetes Obes Metab.

[5] Rasmussen Sara H., Shrestha Sarita, Bjerregaard Lise G., Ängquist Lars H., Baker Jennifer L., Jess Tine, Allin Kristine H. (2018). Antibiotic exposure in early life and childhood overweight and obesity: A systematic review and meta-analysis. Diabetes, Obesity and Metabolism.

[6] Cassidy-Bushrow AE (2017). Prenatal antimicrobial use and early-childhood body mass index. International Journal Of Obesity.

[7] Mor A (2015). Prenatal exposure to systemic antibacterials and overweight and obesity in Danish schoolchildren: a prevalence study. Int J Obes (Lond).

[8] Mueller NT (2015). Prenatal exposure to antibiotics, cesarean section and risk of childhood obesity. Int J Obes (Lond).

[9] Poulsen MN (2017). Associations of prenatal and childhood antibiotic use with child body mass index at age 3 years. Obesity (Silver Spring).

[10] Wang Bin, Liu Jihong, Zhang Yongjun, Yan Chonghuai, Wang Hui, Jiang Fan, Li Fei, Zhang Jun (2018). Prenatal Exposure to Antibiotics and Risk of Childhood Obesity in a Multicenter Cohort Study. American Journal of Epidemiology.

[11] Morgen C S, Ängquist L, Baker J L, Andersen A M N, Michaelsen K F, Sørensen T I A (2017). Prenatal risk factors influencing childhood BMI and overweight independent of birth weight and infancy BMI: a path analysis within the Danish National Birth Cohort. International Journal of Obesity.

[12] Nybo Andersen AM, Olsen J (2002). Do interviewers’ health beliefs and habits modify responses to sensitive questions? A study using data Collected from pregnant women by means of computer-assisted telephone interviews. Am J Epidemiol.

[13] Olsen J (2001). The Danish National Birth Cohort–its background, structure and aim. Scand J Public Health.

[14] Kildemoes HW, Sorensen HT, Hallas J (2011). The Danish National Prescription Registry. Scand J Public Health.

[15] Andersen, C. *Validation of the anthropometric data in the 7-year follow-up. Internet*: http://www.ssi.dk/English/RandD/Research%20areas/Epidemiology/DNBC/Publications%20on%20Background%20and%20Methods/Validation%20of%20height%20weight%20and%20waist%20circumference.aspx.

[16] Cole TJ (1990). The LMS method for constructing normalized growth standards. Eur J Clin Nutr.

[17] Cole TJ, Green PJ (1992). Smoothing reference centile curves: the LMS method and penalized likelihood. Stat Med.

[18] Cole TJ, Bellizzi MC, Flegal KM, Dietz WH (2000). Establishing a standard definition for child overweight and obesity worldwide: international survey. BMJ.

[19] Cole TJ, Flegal KM, Nicholls D, Jackson AA (2007). Body mass index cut offs to define thinness in children and adolescents: international survey. BMJ.

[20] Nohr EA (2005). Prepregnancy obesity and fetal death: a study within the Danish National Birth Cohort. Obstet Gynecol.

[21] Knudsen LB, Olsen J (1998). The Danish Medical Birth Registry. Dan Med Bull.

[22] Ajslev TA, Andersen CS, Gamborg M, Sorensen TI, Jess T (2011). Childhood overweight after establishment of the gut microbiota: the role of delivery mode, pre-pregnancy weight and early administration of antibiotics. Int J Obes (Lond).

[23] Stokholm J (2016). Cesarean section changes neonatal gut colonization. J Allergy Clin Immunol.

[24] Backhed F (2015). Dynamics and Stabilization of the Human Gut Microbiome during the First Year of Life. Cell Host Microbe.

[25] Yassour M (2016). Natural history of the infant gut microbiome and impact of antibiotic treatment on bacterial strain diversity and stability. Sci Transl Med.

[26] Azad MB (2016). Impact of maternal intrapartum antibiotics, method of birth and breastfeeding on gut microbiota during the first year of life: a prospective cohort study. BJOG.

[27] Allin KH, Nielsen T, Pedersen O (2015). Mechanisms in endocrinology: Gut microbiota in patients with type 2 diabetes mellitus. Eur J Endocrinol.

[28] Cox LM (2014). Altering the intestinal microbiota during a critical developmental window has lasting metabolic consequences. Cell.

[29] Jess T (2014). Microbiota, antibiotics, and obesity. N Engl J Med.

[30] Matthiessen J (2014). Trends in overweight and obesity in Danish children and adolescents: 2000–2008–exploring changes according to parental education. Scand J Public Health.

[31] Li D-K, Chen H, Ferber J, Odouli R (2017). Infection and antibiotic use in infancy and risk of childhood obesity: a longitudinal birth cohort study. The Lancet Diabetes & Endocrinology.

