# Supplementary material for: Antibiotic use during pregnancy and childhood overweight: A population-based nationwide cohort study
Source: Sci Rep. 2019 Aug 8;9:11528. doi: 10.1038/s41598-019-48065-9 (PMC6687733; doi:10.1038/s41598-019-48065-9)
Supplement: Supplementary file 1 — Supplementary Material [file 41598_2019_48065_MOESM1_ESM.pdf]

Supplementary Material

Antibiotic use during pregnancy and childhood overweight: A population-based nationwide cohort study

Tine Jess, Camilla S Morgen, Maria C Harpsøe, Thorkild IA Sørensen, Teresa A Ajslev, Julie C Antvorskov, Kristine H Allin

Supplementary Figure 1. Flow chart describing the sampling of the study population

Supplementary Table 1. Categorization of antibiotics into narrow- and broad-spectrum antibiotics

Supplementary Table 2. Characteristics of the population according to whether childhood BMI information was available

Supplementary Table 3. Use of antibiotics in the study population

Supplementary Table 4. Characteristics of the study population according to number of antibiotic courses during pregnancy

Supplementary Table 5. Effects of adjustment for individual covariates included in the multivariable-adjusted model

Supplementary Table 6. Association between antibiotics during pregnancy and obesity in childhood

Supplementary Table 7. Odds of overweight at age seven years according to exposure to antibiotics with and without childhood antibiotics in the model

Supplementary Table 8. Characteristics of the study population according to whether information on BMI was available at age 11 years

Supplementary Figure 1. Flow chart describing the sampling of the study population

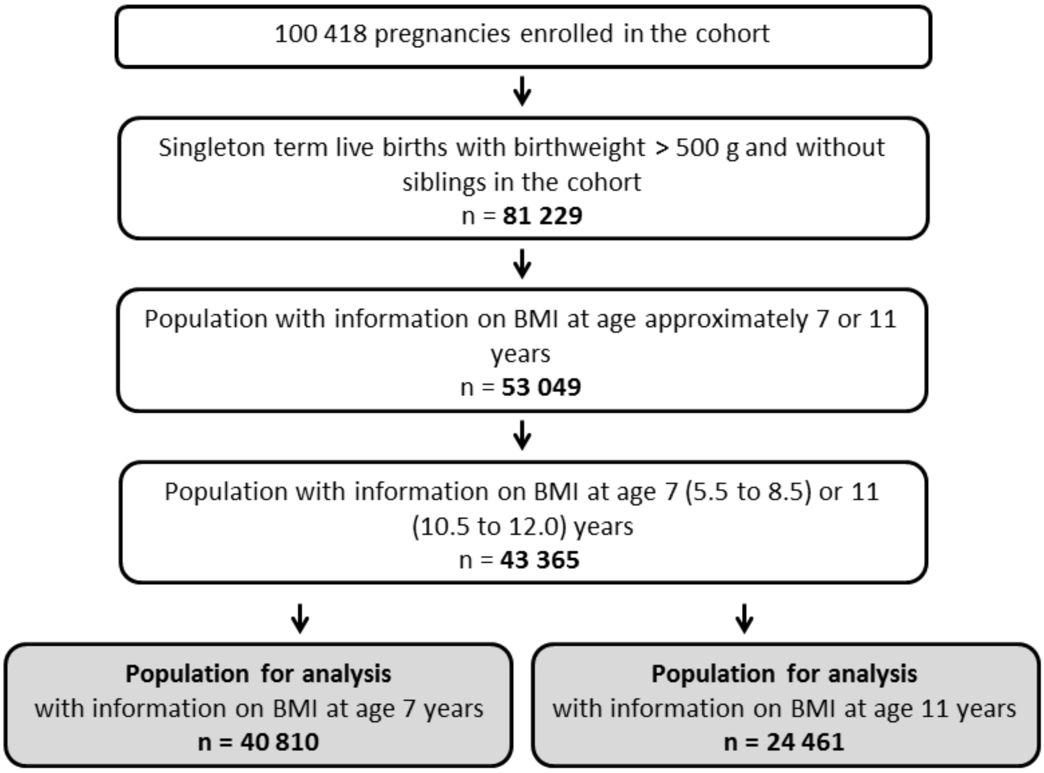

Age- and sex-specific BMI z-scores and overweight at age seven and 11 years were used as outcomes in the study. At age seven years, these were defined for children with BMI measured at age 5.0 to 8.5 years. To avoid influences of puberty at age 11 years, age- and sex-specific BMI z-scores and overweight at age 11 years were defined for children with BMI measured at age 10.5 to 12.0 years.

Supplementary Table 1. Categorization of antibiotics into narrow- and broad-spectrum antibiotics

ATC code	Name	Narrow- or broad-spectrum
J01CA08	Pivmecillinam	Narrow
J01CE02	Phenoxymethylpenicillin	Narrow
J01CE09	Procaine benzylpenicillin	Narrow
J01CF01	Dicloxacillin	Narrow
J01FA01	Erythromycin	Narrow
J01FA02	Spiramycin	Narrow
J01CF05	Flucloxacillin	Narrow
J01FA06	Roxithromycin	Narrow
J01FA09	Clarithromycin	Narrow
J01FA10	Azithromycin	Narrow
J01FF01	Clindamycin	Narrow
J01CA01	Ampicillin	Broad
J01CA02	Pivampicillin	Broad
J01CA04	Amoxicillin	Broad
J01CA06	Bacampicillin	Broad
J01CR02	Amoxicillin and enzyme inhibitor	Broad
J01EA01	Trimethoprim	Broad
J01EB02	Sulfamethizole	Broad
J01EE01	Sulfamethoxazole and trimethoprim	Broad

Supplementary Table 2. Characteristics of the population according to whether childhood BMI information was available

		BMI information at age seven and/or 11 years		
		Not available / not included in the study (n=37 864)	Available / included in the study (n=43 365)	
	**n**	**Mean ± SD or n (%)**	**Mean ± SD or n (%)**	***P* value**
**Antibiotics during pregnancy**, n (%)	81,229	10,327 (27.3)	10,748 (24.8)	<0.001
**Maternal age at birth,** years	81,229	29.6 ± 4.4	30.2 ± 4.2	<0.001
**Maternal pre-pregnancy BMI**, kg/m^2	74,734	23.8 ± 4.5	23.4 ± 4.0	<0.001
**Maternal diabetes**, n (%)	74,370	337 (1.0)	374 (0.9)	0.04
**Smoking in pregnancy**	75,810			<0.001
Non-smokers		24,022 (70.2)	31,845 (76.6)	
1-10 cigarettes per day		7568 (22.1)	7573 (18.2)	
>10 cigarettes per day		2637 (7.7)	2165 (5.2)	
**Parity ≥ 1**, n (%)	75,961	17,252 (50.3)	20,978 (50.4)	0.88
**Family education/occupational class**, n (%)	72,030			<0.001
Highest level		20,862 (64.6)	28,027 (70.5)	
Middle level		10,118 (31.3)	10,684 (26.9)	
Lowest level		1311 (4.1)	1028 (2.6)	
**Gestational age at birth**, days	81,229	281.6 ± 9.0	281.9 ± 8.9	<0.001
**Child gender, girls**, n (%)	81,228	18,586 (49.1)	21,212 (48.9)	0.62
**Weekly gestational weight gain**, kg	60,942	0.38 ± 0.2	0.38 ± 0.1	0.004
**Paternal BMI**, kg/m^2	54,816	25.3 ± 3.2	25.1 ± 3.1	<0.001
**Birth weight**, kg	81,229	3.6 ± 0.5	3.6 ± 0.5	<.0001
**Cesarean section**, n (%)	81,102	5576 (14.8)	6005 (13.9)	<0.001
**Breastfeeding**, n (%)	52,080			<0.001
0.0-19.9 weeks		7552 (34.9)	7991 (26.3)	
20.0-31.9 weeks		3767 (17.4)	5521 (18.1)	
32.0-39.9 weeks		4289 (19.8)	7009 (23.0)	
40.0-95.0 weeks		6936 (27.9)	9915 (32.6)	

Supplementary Table 3. Use of antibiotics in the study population

	**No. (%)[a]**
**No. of antibiotics courses**	
0	32,617 (75.2)
1	8106 (18.7)
2	1923 (4.4)
≥3	719 (1.7)
**Use of antibiotics according to trimester**	
First trimester	3916 (10.7)
Second trimester	4683 (12.6)
Third trimester	4314 (11.7)
**Type of antibiotics**	
Penicillins; beta-lactam antibacterials (J01C)	7969 (19.6)
Sulfonamides and trimethoprim (J01E)	1571 (4.6)
Macrolides, lincosamides & streptogramins (J01F)	931 (2.8)

[a] Numbers in parentheses are % of the total study population.

Supplementary Table 4. Characteristics of the study population according to number of antibiotic courses during pregnancy

	Number of antibiotics courses				
	0	1	2	≥3	
	Mean ± SD or n (%)	Mean ± SD or n (%)	Mean ± SD or n (%)	Mean ± SD or n (%)	*P* value
**Maternal age at birth,** years	30.2 ± 4.2	30.2 ± 4.3	30.0 ± 4.4	30.1 ± 4.4	0.05
**Maternal pre-pregnancy BMI**, kg/m^2	23.3 ± 3.9	23.6 ± 4.2	24.0 ± 4.6	24.7 ±5.0	<0.001
**Maternal diabetes**, n (%)	254 (0.8)	84 (1.1)	21 (1.1)	15 (2.2)	0.001
**Smoking in pregnancy**					<0.001
Non-smokers	24,332 (77.8)	5740 (73.9)	1309 (71.7)	464 (66.6)	
1-10 cigarettes per day	5487 (17.5)	1544 (19.9)	379 (20.7)	163 (23.4)	
>10 cigarettes per day	1468 (4.7)	488 (6.3)	139 (7.6)	70 (10.0)	
**Parity ≥ 1**, n (%)	15,292 (48.8)	4247 (54.5)	1016 (55.5)	423 (60.5)	<0.001
**Family education/occupational class**, n (%)					<0.001
Highest level	21,465 (71.7)	5047 (68.1)	1148 (65.8)	367 (56.4)	
Middle level	7786 (26.0)	2118 (28.6)	525 (30.1)	255 (39.2)	
Lowest level	684 (2.3)	243 (3.3)	72 (4.1)	29 (4.5)	
**Gestational age at birth**, days	281.9 ± 8.8	282.0 ± 8.9	281.6 ± 9.0	281.8 ± 9.4	0.42
**Child gender, girls**, n (%)	15,983 (49.0)	3980 (49.1)	921 (47.9)	328 (45.6)	0.25
**Weekly gestational weight gain**, kg	0.4 ± 0.1	04 ± 0.1	0.4 ± 0.1	0.4 ±0.2	0.03
**Paternal BMI**, kg/m^2	25.0 ± 3.1	25.2 ± 3.1	25.3 ± 3.2	25.2 ± 3.2	<0.001
**Birth weight**, kg	3.6 ± 0.5	3.6 ± 0.5	3.6 ± 0.5	3.7 ± 0.5	0.34
**Cesarean section**, n (%)	4516 (13.9)	1111 (13.7)	277 (14.4)	101 (14.1)	0.88
**Breastfeeding**, n (%)					<0.001
0.0-19.9 weeks	5665 (24.7)	1693 (30.1)	457 (34.6)	176 (35.2)	
20.0-31.9 weeks	4196 (18.3)	993 (17.6)	233 (17.6)	99 (19.8)	
32.0-39.9 weeks	5421 (23.6)	1249 (22.2)	254 (19.2)	85 (17.0)	
40.0-95.0 weeks	7702 (33.5)	1696 (30.1)	377 (28.5)	140 (28.0)	

Supplementary Table 5. Effects of adjustment for individual covariates included in the multivariable adjusted model

	**OR (95% CI)**
**Overweight at age seven years**	
Unadjusted	1.20 (1.11-1.30)
Univariable adjustment for	
Maternal age at birth	1.20 (1.11-1.30)
Maternal pre-pregnancy BMI	1.11 (1.03-1.21)
Maternal diabetes	1.20 (1.11-1.30)
Smoking in pregnancy	1.17 (1.08-1.26)
Parity	1.19 (1.10-1.28)
Family education/occupational class	1.17 (1.08-1.27)
Gestational age at birth	1.20 (1.11-1.30)
Child gender	1.20 (1.11-1.30)
**Overweight at age 11 years**	
Unadjusted	1.27 (1.14-1.41)
Univariable adjustment for	
Maternal age at birth	1.27 (1.14-1.41)
Maternal pre-pregnancy BMI	1.12 (1.00-1.25)
Maternal diabetes	1.26 (1.14-1.40)
Smoking in pregnancy	1.23 (1.10-1.37)
Parity	1.25 (1.13-1.39)
Family education/occupational class	1.25 (1.12-1.39)
Gestational age at birth	1.27 (1.14-1.41)
Child gender	1.27 (1.14-1.41)

Supplementary Table 6. Association between antibiotics during pregnancy and obesity in childhood

	OR (95% CI)	*P* value
**Seven years**		
Unadjusted	1.39 (1.15-1.68)	<0.001
Multifactor-adjusted	1.13 (0.93-1.38)	0.21
**11 years**		
Unadjusted	1.56 (1.13-2.17)	<0.001
Multifactor-adjusted	1.06 (0.75-1.50)	0.75

Supplementary Table 7. Odds of overweight at age seven years according to exposure to antibiotics with and without childhood antibiotics in the model

		Multivariable-adjusted		Multivariable-adjusted, including childhood antibiotics	
	**n**	**OR (95% CI)**	***P* value**	**OR (95% CI)**	***P* value**
**Overweight at age seven years**					
Narrow- or broad-spectrum antibiotics					
No antibiotics	27,606	1		1	
Broad-spectrum antibiotics	3067	1.20 (1.07-1.36)	0.002	1.20 (1.06-1.35)	0.003
Type of broad-spectrum antibiotics					
No antibiotics	27,606	1		1	
Ampicillin	1094	1.27 (1.05-1.53)	0.01	1.27 (1.05-1.53)	0.001
Amoxicillin	574	1.56 (1.23-1.97)	<0.001	1.55 (1.22-1.97)	<0.001

Childhood antibiotics were defined as exposure to antibiotics within the first 6 months of life.

Supplementary Table 8. Characteristics of the study population according to whether information on BMI was available at age 11 years

		BMI information		
		Not available at 11 years (n=18 904)	Available at 11 years (n=24 461)	
	n	Mean ± SD or n (%)	Mean ± SD or n (%)	*P* value
**Maternal age at birth,** years	43,365	30.0 ± 4.2	30.4 ± 4.2	<0.001
**Maternal pre-pregnancy BMI**, kg/m^2	41,057	23.5 ± 4.2	23.3 ± 3.9	<0.001
**Maternal diabetes**, n (%)	41,871	197 (1.1)	177 (0.8)	0.002
**Smoking in pregnancy**	41,583			<0.001
Non-smokers		13,315 (73.5)	18,530 (79.0)	
1-10 cigarettes per day		3706 (20.4)	3867 (16.5)	
>10 cigarettes per day		1107 (6.1)	1058 (4.5)	
**Parity ≥ 1**, n (%)	41,662	9496 (52.3)	11,482 (48.9)	<0.001
**Family education/occupational class**, n (%)	39,739			<0.001
Highest level		11,530 (66.6)	16,497 (73.6)	
Middle level		5208 (30.1)	5476 (24.4)	
Lowest level		571 (3.3)	457 (2.0)	
**Gestational age at birth**, days	43,365	281.9 ± 8.9	281.9 ± 8.9	0.96
**Child gender, girls**, n (%)	43,365	8931 (47.2)	12,281 (50.2)	<0.001
**Weekly gestational weight gain**, kg	34,994	0.38 ± 0.1	0.37 ± 0.1	0.002
**Paternal BMI**, kg/m^2	32,999	25.1 ± 3.1	25.0 ± 3.1	0.002
**Birth weight**, kg	43,365	3.6 ± 0.5	3.6 ± 0.5	0.95
**Cesarean section**, n (%)	43,365	2665 (13.6)	3440 (14.1)	0.14
**Breastfeeding**, n (%)	30,436			<0.001
0.0-19.9 weeks		3667 (29.1)	4324 (24.3)	
20.0-31.9 weeks		2316 (18.4)	3205 (18.0)	
32.0-39.9 weeks		2719 (21.6)	4290 (24.1)	
40.0-95.0 weeks		3907 (31.0)	6008 (33.7)	
